# Origins and Evolution of Human Tandem Duplicated Exon Substitution Events

**DOI:** 10.1093/gbe/evac162

**Published:** 2022-11-08

**Authors:** Laura Martinez-Gomez, Daniel Cerdán-Vélez, Federico Abascal, Michael L Tress

**Affiliations:** Bioinformatics Unit, Spanish National Cancer Research Centre (CNIO), C. Melchor Fernandez Almagro, 3, 28029 Madrid, Spain; Bioinformatics Unit, Spanish National Cancer Research Centre (CNIO), C. Melchor Fernandez Almagro, 3, 28029 Madrid, Spain; Somatic Evolution Group, Wellcome Sanger Institute, Wellcome Genome Campus, Hinxton, Cambridgeshire CB10 1SA, United Kingdom; Bioinformatics Unit, Spanish National Cancer Research Centre (CNIO), C. Melchor Fernandez Almagro, 3, 28029 Madrid, Spain

**Keywords:** alternative splicing, exon duplication, function, proteomics, tissue specificity, ion channels

## Abstract

The mutually exclusive splicing of tandem duplicated exons produces protein isoforms that are identical save for a homologous region that allows for the fine tuning of protein function. Tandem duplicated exon substitution events are rare, yet highly important alternative splicing events. Most events are ancient, their isoforms are highly expressed, and they have significantly more pathogenic mutations than other splice events.

Here, we analyzed the physicochemical properties and functional roles of the homologous polypeptide regions produced by the 236 tandem duplicated exon substitutions annotated in the human gene set. We find that the most important structural and functional residues in these homologous regions are maintained, and that most changes are conservative rather than drastic. Three quarters of the isoforms produced from tandem duplicated exon substitution events are tissue-specific, particularly in nervous and cardiac tissues, and tandem duplicated exon substitution events are enriched in functional terms related to structures in the brain and skeletal muscle.

We find considerable evidence for the convergent evolution of tandem duplicated exon substitution events in vertebrates, arthropods, and nematodes. Twelve human gene families have orthologues with tandem duplicated exon substitution events in both *Drosophila melanogaster* and *Caenorhabditis elegans*. Six of these gene families are ion transporters, suggesting that tandem exon duplication in genes that control the flow of ions into the cell has an adaptive benefit. The ancient origins, the strong indications of tissue-specific functions, and the evidence of convergent evolution suggest that these events may have played important roles in the evolution of animal tissues and organs.

**Significance**

Tandem duplicated exon substitutions are a highly important yet little studied class of alternative splice events. These events are ancient, highly tissue-specific, and have evolved independently across orthologous genes in a range of metazoa. Our results suggest that tandem duplicated exon substitutions were important in the evolution of specific tissues and organs.

## Introduction

The human reference genome is annotated with fewer than 20,000 coding genes. GENCODE (Frankish et al. 2021) v40 annotated 19,988 coding genes, and according to Ensembl (Howe et al. 2021), 662 of these are read-through genes; genes that are annotated solely with transcripts that read through from one gene to a neighbor (Abascal et al. 2018). There is no evidence that read-through transcripts can produce stable protein products, and they will soon no longer be annotated as protein coding. However, the number of protein isoforms produced from the reference gene set will be greater than 19,336 because coding genes can generate multiple alternatively spliced mRNA transcripts (Bush et al. 2017). Alternative splicing has been detected in almost all multi-exon coding genes (Wang et al. 2008). The Ensembl/GENCODE and RefSeq (O’leary et al. 2016) annotations of the human gene set predict more than 137,000 isoforms between them, although this includes many unfinished transcripts fragments (Ensembl/GENCODE) and computer predictions (RefSeq).

The clear evidence of alternative splicing at the transcript level has led to the assumption that alternative splicing underpins cellular protein diversity (Graveley 2001) and caused some to argue that alternative splicing is one of the main drivers of mammalian complexity (Kim et al. 2007; Chen et al. 2014).

Two facts about alternative splicing are clear. Firstly, the experimental support for the hypothesis that most coding genes have a single main protein isoform is overwhelming (Ezkurdia et al. 2015a; Pozo, Martinez Gomez, et al. 2022; Pozo, Rodriguez, et al. 2022). In humans, we have shown that MANE Select transcripts (Morales et al. 2022) and APPRIS principal isoforms (Rodriguez et al. 2022) are the best predictors of this main splice variant (Pozo, Martinez Gomez, et al. 2022; Pozo, Rodriguez, et al. 2022).

Secondly, all researchers have recognized that there are a number of well-known functionally important alternative splice isoforms, and these isoforms have been catalogued in multiple reviews (Tress et al. 2017a; Bhuiyan, et al. 2018; Rodriguez et al. 2020; Marasco and Kornblihtt 2022; Wright et al. 2022). However, despite the existence of these catalogues, the actual proportion of alternative transcripts that are translated into functionally important proteins is still unknown (Bush et al. 2017; Tress et al. 2017a, 2017b; Bhuiyan, et al. 2018; Wright et al. 2022).

Results from large-scale proteomics experiments (Ezkurdia et al. 2015a; Rodriguez et al. 2020) suggest that most alternative transcripts are either translated in limited circumstances, in much lower quantities or have much shorter half-lives than those translated from the main cellular transcript. Although the relative absence of proteomics evidence is not conclusive proof that most alternative isoforms have little functional relevance, the evidence from human genetic variation studies is more damning. Alternative exons are not under selective pressure (Liu and Lin 2015; Tress et al. 2017a; Pozo, Martinez Gomez, et al. 2022). There is also little clinical evidence for the importance of alternative protein isoforms; just 0.05% of validated ClinVar (Landrum et al. 2018) pathogenic mutations affect alternative proteins (Pozo, Rodriguez, et al. 2022).

Part of the reason that most alternative isoforms appear to have little or no support for their functional relevance is likely to be the relative age of the splice events. Three quarters of annotated alternative transcripts in the human gene set are primate-derived (Rodriguez et al. 2020), and very few of the isoforms translated from these novel transcripts are detected at the protein level. Even when they are translated, there is little evidence that these splice variants have a cellular role (Martinez-Gomez et al. 2020; Rodriguez et al. 2020).

The more conserved the alternative splice variant, the more likely it is to be functionally important (Rodriguez et al. 2020). In the human gene set, some 5% of annotated splice events have a last common ancestor in fish (Gómez et al. 2021), and almost one in three of these ancient alternative splice events involves tandem duplicated exons that are spliced in a mutually exclusive manner (Gómez et al. 2021). These splice events produce two proteins that are identical save for a single unique but homologous region of amino acids.

First characterized by Kondrashov and Koonin (2001), mutually exclusively spliced tandem duplicated exons were initially thought to be involved in as many as 20% of alternative splice events (Letunic et al. 2002). However, while tandem duplication is a common process, the mutually exclusive splicing of the duplicated exons is not. An initial analysis of the human genome predicted 629 mostly homologous mutually exclusively spliced alternative splicing events (Hatje et al. 2017). However, many of these predictions were not true alternative splice events (Gómez et al. 2021). More recently, Lam et al. (2021) detailed 143 mutually exclusively spliced events in the human genome, limiting themselves to just those events that underwent true mutually exclusive splicing (Pohl et al. 2013). We carried out an in-depth analysis, finding that there are just 236 annotated tandem duplicated exon events in the human gene set (Gómez et al. 2021). This means that tandem duplicated exon substitutions make up just 0.3% of all currently annotated splice events (Gómez et al. 2021).

Although tandem exon duplication events and mutually exclusively spliced events are often mentioned together and share some characteristics, they should not be confused. Firstly, not all mutually exclusively spliced events evolved from tandem exon duplications. Many mutually exclusively spliced events involve apparently nonhomologous exons. Secondly, not all tandem exon duplication events are mutually exclusively spliced, since mutually exclusive splicing cannot involve 3′ or 5′ exons (Pohl et al. 2013; Hatje et al. 2017) and in the strictest definition of mutually exclusive splicing, the duplicated exons cannot be annotated in the same transcript (Pohl et al. 2013; Lam et al. 2021). However, it turns out that the mechanism behind a splice event is unrelated to its functional importance. Tandem exon duplication events, which produce isoforms with homologous substitutions, have abundant support for their functional importance (Abascal et al. 2015; Abascal, Tress, et al. 2015; Rodriguez et al. 2020; Gómez et al. 2021; Lam et al. 2021). Mutually exclusively spliced nonhomologous exons do not (Gómez et al. 2021).

Tandem exon duplication substitution events are of ancient origin (Abascal et al. 2015; Gómez et al. 2021). More than 90% of the tandem exon duplication events annotated in the human genome have orthologues in bony fish, most can trace their evolution back to the earliest vertebrates (Gómez et al. 2021), and 21 events annotated in the human genome even have an orthologous event in invertebrate species (Gómez et al. 2021). They are also highly enriched in large-scale proteomics experiments (Abascal et al. 2015; Gómez et al. 2021), and even capture 20 times as many pathogenic mutations as other splice events (Gómez et al. 2021). Tandem exon duplication events are clearly the most important class of alternative splice events.

One of the earliest papers on tandem duplicated exon substitution events (Copley 2004) found three events that appeared to have arisen independently in human and *Drosophila melanogaster* orthologues. Curiously, the events were in three different ion channel families (two voltage-gated ion channels and a calcium-activated potassium channel). Although this was only a small sample, the evidence for the convergent evolution of tandem duplicated exon events suggested that tandem exon duplications might provide advantages in certain protein families.

Lam et al. (2021) carried out an in-depth molecular characterization of mutually exclusively spliced homologous exons in five different species. They found that regions translated from homologous mutually exclusively spliced exons were enriched in surface residues, and were therefore likely to influence binding to ligands and to other proteins. Within these regions, residues that were distinct between the two isoforms tended to be found even more often on the protein surface than residues that were unchanged between the two isoforms.

In this paper, we investigate the physicochemical properties, tissue specificity and functional role of the distinct homologous polypeptide regions generated by the 236 tandem duplicated exon substitution events in our curated set. These regions, referred to in this paper as unique homologous polypeptide regions (UHP regions for short), are enriched in solvent accessible residues. Residues in functional domains and those that bind ligands are significantly more conserved between UHP regions, whereas residues in predicted disordered regions are not conserved between regions. Most substitutions are conservative, suggesting that there are evolutionary pressures to maintain the core protein function in both isoforms. Tandem duplicated exon events are ancient, and there is considerable evidence for the convergent evolution of these splice variants in vertebrate and invertebrate species. Genes with tandem duplicated exon substitution events are highly enriched in tissue-specific terms, and in ion channels and other transmembrane proteins, and their isoforms have elevated levels of tissue specificity. This raises the intriguing possibility that these splice events may have played roles in the evolution of metazoan organs and tissues.

## Results

### Tandem Duplicated Exon Substitutions

The set of tandem duplicated exon events analyzed in this paper was generated by manual curation from the Ensembl/GENCODE human gene set (Frankish et al. 2021). Duplicated exons had to be consecutive in sequence, and be annotated more often than not in distinct coding transcripts, whereas the translated polypeptide regions had to have evidence of homology and be at least seven amino acids long. The manual curation is described in detail in a previous paper (Gómez et al. 2021). We identified 236 tandem duplicated exon substitutions across 214 coding genes. Much of the analysis was carried out on the UHP regions produced by the duplicated exons.

### The Position of Tandem Duplicated Exon Substitutions

Exactly half of the UHP regions from the 236 tandem duplicated exon events are produced from internal, mutually exclusively spliced CDS. Of the remaining UHP regions, 40 involve substitutions of N-terminal polypeptides, and 77 involve the swap of one C-terminal UHP region for another. The final event (in *DUSP13*) covers the whole protein.

Although there are more internal mutually exclusively spliced coding exons (CDS), UHP regions are enriched in N- and C-terminal substitutions. This is because internal CDS are much more numerous than 5′ or 3′ CDS. In fact, internal CDS that are at least 21 bases in length outnumber 5′ or 3′ terminal CDS by 15:1 over the 214 genes in our set of tandem exon duplication events. So, if the process of duplicating and becoming mutually exclusively spliced was random, we would expect that 205.9 of the 236 tandem duplicated exon events would involve internal CDS. There are only 118 in our set. At the same time, we would expect to detect just 15 events that involved each of the terminal CDS, so N-terminal (40 events) and especially C-terminal substitutions (77 events) are highly enriched in our set.

### Tandem Duplicated Exon Substitutions and Protein 3D Structure

We mapped the UHP regions to the EBI-AlphaFold structures (Varadi et al. 2022) for representative human proteins made available by UniProtKB (The UniProt Consortium 2021). We were able to use the models to analyze UHP regions from 231 of the 236 genes (see methods section). Lam et al. (2021) showed that residues in UHP regions tended to be more exposed to solvent than residues in the rest of the protein. We confirmed their results with the EBI-AlphaFold models. A total of 69.8% of residues in UHP regions are classified as surface accessible below a DSSP cut-off of 0.2, compared with just 64.1% of residues not in UHP regions in the same proteins. The difference is statistically significant (χ^2^ test *P* < 0.00001).

**Fig. 1.**
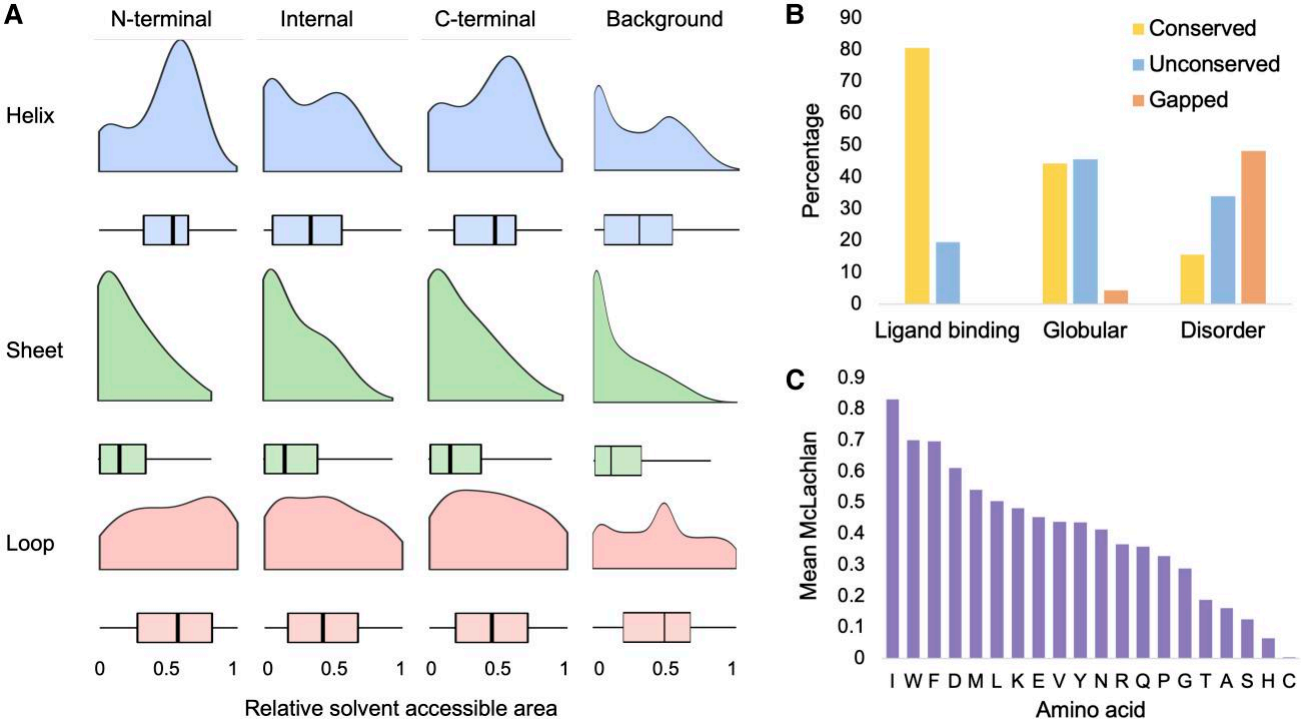
Physicochemical properties of resides in UHP regions. In (*A*), the distribution of relative solvent accessible area (RSA) for amino acid residues in loops, sheets, and helices in three distinct types of UHP regions (N-terminal, Internal, and C-terminal), compared with the background distribution form the whole set of AlphaFold models. Panel (*B*) shows the percentage of ligand binding, ordered (globular), and disordered residues that are conserved between two UHP regions, that change, or that align to a gap. In (*C*), the average gain in McLachlan Matrix score for each type of amino acid in the alignments of the UHP regions. The amino acids are taken from the UHP region that is modeled by AlphaFold in each case. The gain is calculated by subtracting the average McLachlan Matrix score from the average score of the substitutions in the UHP regions.

We find that the enrichment in solvent-exposed residues in UHP regions is almost entirely due to the overabundance of N- and C-terminal UHP regions. While UHP regions produced by internal CDS have a similar distribution of DSSP accessibility scores as the rest of protein, residues in C- and N-terminal UHP regions are clearly more solvent exposed (fig. 1). This exposure is particularly noticeable among residues predicted as helices by EBI-AlphaFold (fig. 1). This is perhaps not surprising, since many N-terminal and C-terminal regions will be made up of signal peptides, signal anchors, single transmembrane helices, coiled coils, and disordered regions.

### Disorder within UHP Regions

By mapping the UHP regions to the EBI-AlphaFold structures and Pfam functional domains (El-Gebali et al. 2019), we found that 204 UHP regions coincided at least in part with either a structural or a functional domain. In total, 87 UHP regions overlapped regions defined as disordered from the EBI-AlphaFold predictions (at least 3 consecutive amino acids with pLDDT ≤ 50), so some UHP regions overlapped with both Pfam domains and disordered residues. Where UHP regions overlapped both Pfam domains and predicted disordered residues, the residues coinciding with the Pfam domains were considerably more conserved.

Curiously, there is a slight tendency for UHP regions not to coincide with predicted disordered regions. Just 23.7% of residues in the UHP regions are predicted to be disordered from the EBI-AlphaFold models, compared with 25.4% of residues in those genes that harbor tandem duplicated exon substitution events, and to 28.3% of all residues in UniProtKB display isoforms. The fact that UHP regions are less disordered may simply be because homology is harder to detect between two disordered UHP regions. There are almost certainly tandem duplicated exon substitutions events that produce disordered UHPs, but that have diverged so much that they are beyond detection.

We generated alignments between the UHP regions for each event, and used the EBI-AlphaFold models to analyze the relationship between disorder (residues with a pLDDT score lower than 50) and conservation of residues in the alignments. Just 15.6% of predicted disordered residues were conserved in the alignments, compared with 44.3% of nondisordered residues (fig. 1). Predicted disordered residues are almost three times less conserved. In fact, almost half of the disordered residues aligned to gaps (48.2%). Within UHP regions, conserved functional and structural domains are substantially more conserved than disordered regions.

### Amino Acid Changes in UHP Regions

Lam et al. found that residues that changed between UHP regions tended to be more exposed to solvent. We also find that residues in the UHP regions that change between two isoforms are much more likely to be accessible to solvent (74.1%) than residues that are conserved between the two UHP regions (65.1%). This is to be expected; buried residues will always be more conserved because of their importance to folding and stability (Goldman et al. 1998).

However, we could not confirm the finding that most residues that changed between UHP regions undergo drastic changes, leading to large changes in function between the distinct protein isoforms (Lam et al. 2021). Instead, we find that amino acid residue changes within the UHP regions are more conservative than would be expected by chance. We calculated a measure of conservation using the scores from the McLachlan matrix (McLachlan 1971). The average of the scores derived from the McLachlan matrix for all amino acids that changed in aligned UHP regions was 3.43, which is considerably higher than the average McLachlan substitution (2.4), and two points higher than the Lam et al. set. The amino acid residues that undergo the least changes are generally the large hydrophobic amino acids (isoleucine, tryptophan, phenylalanine, leucine; fig. 1 and supplementary fig. S1, Supplementary Material online). The fact that residue changes are conservative, particularly among large hydrophobic amino acids, suggests that the duplicated exons are under strong selective pressure to maintain protein 3D structure and core function of the UHP regions.

### Functional Residues and Homologous Exons

We find that the UHP regions in our set tend not to overlap ligand binding residues. UHP regions comprise 14% of all residues in genes for which *firestar* (Lopez et al. 2011) predicts ligand binding sites, but the UHP regions overlap just 10.7% of the predicted functional residues. This difference is significant (Fisher’s exact test *P* < 0.00001), and is most likely also due to the enrichment in N- and C-terminal tails in UHPs. N- and C-terminal tails are rich in transmembrane helices, coiled-coil motifs, and disordered residues which almost never coincide with ligand binding residues.

**Fig. 2:**
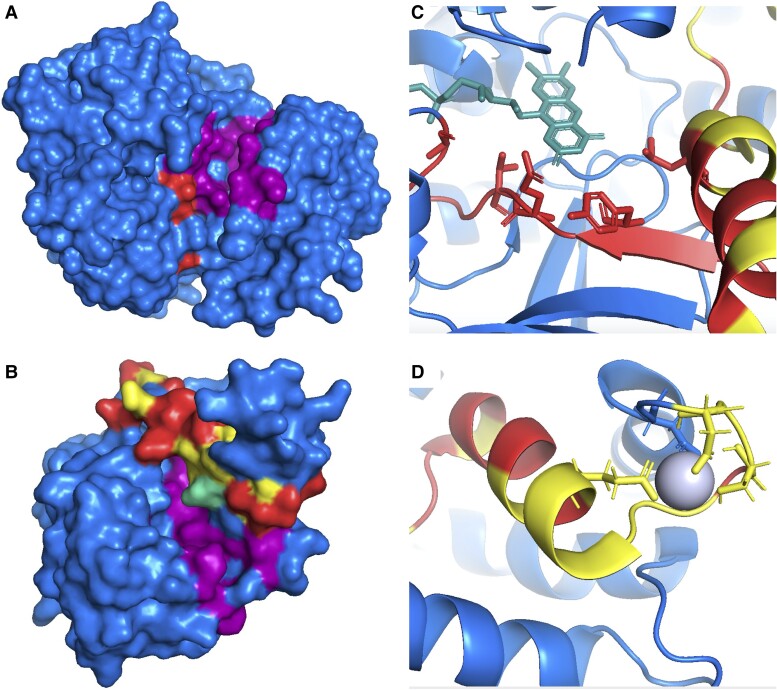
Tandem exon duplications and functional residues. Resolved structures of isoforms of (*A*) *CAMK2D* (PDB (Burley et al. 2017) structure: 6ayw) in spacefill mode showing the protein surface, (*B*) *KHK* (2hqq) in spacefill mode, (*C*) *ACOX1* (7q86) in cartoon mode with ligand binding residues shown as sticks, and (*D*) *ACTN1* (2n8y) also in cartoon mode with ligand binding residues shown as sticks. For all panels, the UHP regions are highlighted in red if the amino acids are conserved between UHP regions, and in yellow when not conserved. Ligand binding regions in panels (*A*) and (*B*) are shown in purple. Ligand binding regions in panels (*C*) and (*D*) are shown as sticks. The teal residue in panel (*B*) is arginine 108, part of both the ligand binding region and the UHP region. The teal structure in panel (*C*) is the bound flavin-adenine dinucleotide ligand. The ball in panel (*D*) is the bound calcium. All images were created with PyMol.

Although UHP regions tend not to overlap with the small ligand binding sites, there are UHP regions that are found adjacent to ligand binding sites, for example in *CAMK2D* and *KHK* (fig. 2). The UHP region in the *CAMK2D* protein crosses from one side of the domain to the other, but there is a cluster of residues right next to the predicted ATP binding site. The UHP region in *KHK* lies right alongside the ATP binding site, yet of all the residues predicted to be in contact with the ligand, only the catalytic arginine (residue 108) falls in the UHP region. This catalytic arginine is conserved in both isoforms.

Where UHP regions do overlap ligand binding residues, these residues are almost always conserved between the two isoforms, with 80.6% of predicted ligand binding residues in UHP regions are maintained, against 53.1% of all other residues in UHP regions in the same proteins (fig. 1*B*). In fact, UHP regions that include predicted ligand binding residues are themselves much more conserved (53.1%) than the UHP regions that do not have ligand binding residues (37.4%). An example is shown in figure 2*C*. In gene *ACOX1*, the UHP region has 55 amino acids. Just over half of the amino acids (28) are conserved between the two UHP regions, including all five of the predicted flavin-adenine dinucleotide binding residues.

In most genes, no more than one or two ligand binding residues differ between the two UHP regions. For example, both genes *PKM* and *PFKP* have a ligand binding residue involved in the allosteric binding of fructose-1,6-bisphosphate that differs between two splice isoforms (Lam et al. 2021). Three genes have lost most or all of their predicted binding residues in one of the two UHP regions. Tandem duplicated exon substitution events affect residues in EF-hand motifs in both *ACTN1* and *ACTN4* (fig. 2*D*), and only one of the two UHP regions maintains the ability to bind calcium (Waites et al. 1992). Again it is calcium binding residues that are not conserved in one of the two UHP regions in *ACAN*.

### Tissue Specificity of UHP Regions

We found validated peptide evidence for 11,898 coding genes from the two large-scale, tissue-based proteomics analyses, 61.5% of the 19,336 non-read-through genes annotated in the GENCODE gene set. Genes with tandem exon duplication substitution events are apparently more highly expressed than average, because we detected peptides for 176 of the 214 genes (82.2%). We have already demonstrated that there is a strong relationship between gene family age and whether or not a protein is detected in a proteomics experiment (Abascal et al. 2018), and this is almost certainly what is happening here. Genes with tandem exon duplication substitution events are more ancient than the general population of coding genes: 61.9% of all genes in the GENCODE gene set have a last common ancestor that pre-dates the Bilaterian clade, while the same is true for 84.6% of the 214 genes with tandem exon duplication substitution events (181).

We detected peptides that supported both sides of the tandem exon splice event in 70 of the 176 genes (39.8%). Here again, age played a role. We detected peptides for both UHP regions for 15 of the 21 splice events that evolved in the Bilaterian clade, and for more than half of the events that arose in chordates, but only two of the 21 events that evolved after the split from teleost fish had peptides that supported both UHP regions. Another factor was whether or not the protein was an integral transmembrane protein. Transmembrane regions are harder to detect in proteomics experiments. Just 8 of 70 genes with peptide support for both UHP regions were integral transmembrane proteins (11.3%), compared with 31 of the 106 genes with proteomics support, but without peptide evidence for both UHP regions (29.5%).

In total, we found peptide support for 81 tandem exon duplication substitution events (supplementary table S2, Supplementary Material online). Several of the 70 genes have peptide evidence for more than one tandem exon duplication substitution event (we detected peptides for both UHP regions in five different events in *TPM1*, for example). We carried out an analysis of tissue specificity for the 81 events. We found that one or more isoforms were significantly enriched in at least one tissue, or group of tissues, in 57 of these 81 tandem exon duplication substitution events (70.4%).

For the other 24 events, we did not find significant enrichment in any tissue, but 10 of these events had 8 or fewer peptide experiment counts (PECs) for one side of the splice event, suggesting that the proportion of significantly tissue-enriched tandem exon duplication substitution events might be higher with increased peptide coverage.

One group of tissues stands out among isoforms that are tissue-specific at the protein level. More than half of the tissue-specific events, (31) have one isoform that is significantly enriched in nervous tissues, here defined as brain, frontal cortex, spinal cord, and retina. Another 19 isoforms were significantly enriched in heart tissues, and 11 were significantly enriched in hematopoietic cells.

**Fig. 3:**
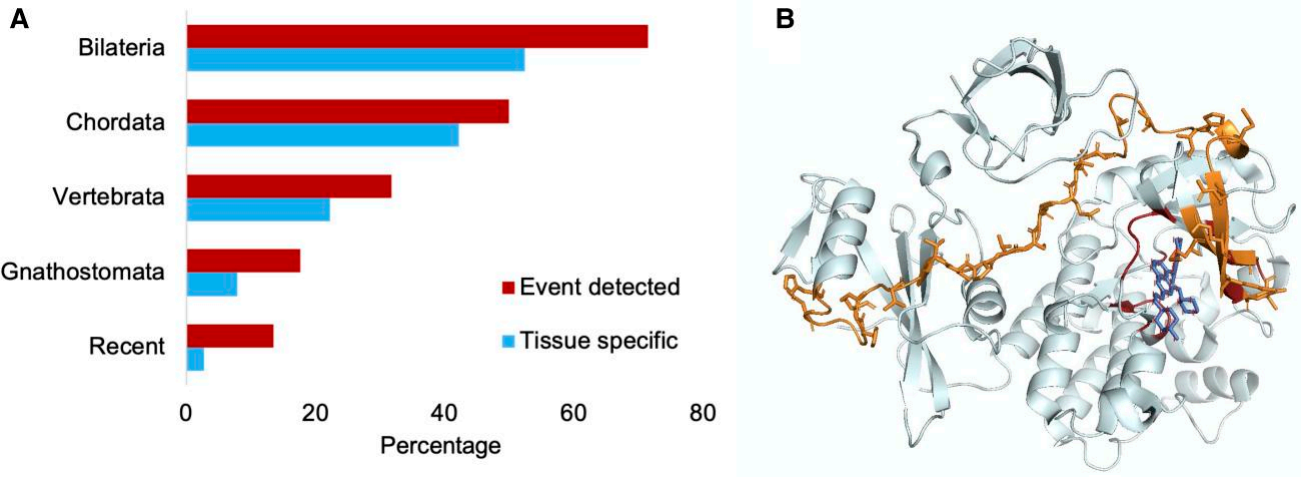
Tissue specificity of UHP regions. In panel (*A*), the percentage of tandem duplicated substitution events for which we detected both UHP regions in large-scale proteomics experiments broken down by the age of the event, and the percentage of tandem duplicated substitution events that were both detected and tissue specific at the protein level. Peptide evidence for both UHP regions was found for almost 80% of Bilaterian events, and more than 50% were also found to be tissue-specific. In panel (*B*), the AlphaFold [47] model of one of the two tissue-specific *FYN* isoforms (FynB in this case). The UHP region that spans the two domains is coloured. The conserved residues in the UHP region that interact with the adenosine triphosphate (ATP) ligand are in red.

The tissue-specific events that we detected all evolved more than 450 million years ago (fig. 3), and most (36) evolved before the divergence of *tunicates* and vertebrates, more than 600 million years ago. Among these splice events, tissue-specific splicing was even more prominent; more than 80% of the splice events that evolved prior to the chordate clade were significantly enriched at the protein level.

Apart from the 214 genes annotated with alternatively spliced tandem duplicated exons in GENCODE, there are also a number of tandem duplicated, alternatively spliced 5′ exons that are annotated in distinct genes. The duplicated exons are annotated in distinct genes for historical rather than functional reasons (Gómez et al. 2021). There are five of these “gene clusters”, and we find sufficient peptide evidence to allow us to evaluate tissue-specific splicing in 18 distinct alternatively spliced isoforms from 3 of these clusters (we detected peptide evidence for 20 isoforms, but 2 did not have sufficient PECs). Sixteen of the remaining 18 alternatively spliced isoforms are significantly enriched in one or more tissues or tissue groups.

All eight isoforms detected for the UDP glucuronosyltransferase family 1 cluster are tissue-specific in digestive tissues, in kidney, liver, lung, urinary bladder, or tonsil. Six of the isoforms detected for the protocadherin gamma subfamily cluster are also tissue-specific, in nervous tissues, pituitary, thyroid, or reproductive tissues, while the other two (with just nine and four PECs) might have been tissue-specific if there were more supporting peptides. Unlike the other tissue-specific tandem exon splice events, the multiple events in the protocadherins and in the UDP glucuronosyltransferases evolved in the euteleostomi clade. If we count the gene “clusters” as separate isoforms of the same gene (as we should), then 71 of 99 tandem exon duplication substitution events with the minimum sufficient peptide coverage are tissue-specific at the protein level.

### Tissue Specificity and Subfunctionalization

Gene duplications allow the separation of functions into distinct genes. When genes with tandem exon duplication substitution events duplicate, there are three different possible outcomes for tandem duplicated exons from the events. Firstly, many tandem duplicated exons are conserved in both genes, as is the case with six of the sodium channel alpha subunits in the set of genes with tandem exon duplication substitution events. Another possibility is that one duplication may conserve both exons, whereas the other gene loses one of the tandem duplicated exons. This happened with the duplication that created *PGM1* and *PGM5* (Abascal, Tress, et al. 2015). Here, only *PGM1* maintained both tandem duplicated exons. The final possibility is subfunctionalization (Abascal, Tress, et al. 2015): after duplication, one of the tandem duplicated exons is maintained in one gene, whereas the other exon is maintained in the other gene. This is what happened in the ancestor of the voltage-gated calcium channel genes, *CACNA1F* and *CACNA1S*. The ancestral sequence had three tandem exon duplication substitution events, and *CACNA1C* and *CACNA1D* have retained all three sets of tandem duplicated exons. However, *CACNA1F* and *CACNA1S* are only annotated with one of the two tandem duplicated exons. Curiously, each gene maintained a different exon from all three of the tandem exon duplication substitution events, suggesting that these three splice events may work in concert.

*FYN*, an Src family tyrosine kinase, has a pair of mutually exclusively spliced tandem duplicated exons. Src family tyrosine kinases regulate many signaling pathways and *FYN* itself has been shown to be important in cancer (Elias and Ditzel 2015) and Alzheimer's disease (Matrone et al. 2020). The homologous exons cover a region that includes the linker between the SH2 and kinase domains and the N-terminal of the kinase domain including the ATP binding site (fig. 3). The two isoforms generated from these tandem duplicated exons, FynT and FynB, have different binding affinities for the binding and phosphorylation of KH domain-containing, RNA-binding, signal transduction-associated protein 1 (Brignatz et al. 2009) and their transcripts have sharply different expression patterns; FynB transcripts are expressed ubiquitously, but not in blood or spleen, whereas FynT is highly expressed in blood and spleen, but in low quantities in all other tissues. This pattern is clear at the protein level too (Kim et al. 2014; Rodriguez et al. 2020). In fact, the improper expression of the blood-specific variant in brain tissues may be related to Alzheimer's disease (Low et al. 2021).

All four tyrosine kinases that have evolved as a result of *FYN* duplications (*FGR*, *SRC*, *YES1*, and *FRK*) have undergone subfunctionalization. All have retained just one of the two tandem duplicated exons. Curiously, all four genes have also retained one of the two *FYN* tandem exon expression patterns, though not always the one that corresponds to the *FYN* tandem duplicated exon it has maintained. *FRK* is the only gene to maintain the FynB tandem duplicated exon, the exon from the transcript that is expressed ubiquitously but not in blood or spleen. *FRK* is also expressed ubiquitously, but not at all in blood. Of the three genes that retained the FynT tandem exon (which is expressed specifically in blood and spleen), one (*FGR*) is also expressed almost entirely in blood and spleen, but the other two (*SRC* and *YES1*) are expressed in almost all tissues *apart* from blood and spleen.

### Functional Characterization of Genes with Tandem Exon Duplication Substitution Events

We determined the distribution of GO functional terms (The Gene Ontology Consortium 2019) for the 214 human genes with tandem exon duplication substitution events using DAVID (Sherman et al. 2022). The top 10 terms can be seen in table 1. Two tissue-specific terms stand out, glutamatergic synapse (2.74 × 10^−11^), and Z-disc (7.59 × 10^−10^). Besides those terms, most remaining terms are related to plasma membrane proteins and ion channels (e.g., plasma membrane, 7.57 × 10^−11^; regulation of ion transmembrane transport, 1.44 × 10^−09^; voltage-gated ion channel activity, 2.19 × 10^−06^). Almost half of human genes (47.2%) with tandem exon duplication substitution events are found in the plasma membrane.

**Table 1:** Top GO Terms for Human Genes with Tandem Exon Duplication Substitution Events

GO term	Count	*P*-value	Enrichment	Benjamini
GO:0098978∼glutamatergic synapse	26	8.1 × 10^−14^	6.86	2.74 × 10^−11^
GO:0005886∼plasma membrane	100	4.48 × 10^−13^	1.94	7.57 × 10^−11^
GO:0030018∼Z-disc	16	6.74 × 10^−12^	11.75	7.59 × 10^−10^
GO:0034765∼regulation of ion transmembrane transport	17	9.46 × 10^−13^	11.92	1.44 × 10^−09^
GO:0070062∼extracellular exosome	52	1.29 × 10^−08^	2.31	1.09 × 10^−06^
GO:0003779∼actin binding	20	3.74 × 10^−09^	5.55	1.74 × 10^−06^
GO:0016020∼membrane	56	2.60 × 10^−08^	2.16	1.75 × 10^−06^
GO:0005244∼voltage-gated ion channel activity	10	9.42 × 10^−09^	16.22	2.19 × 10^−06^
GO:0016021∼integral component of membrane	91	7.27 × 10^−08^	1.66	4.10 × 10^−06^
GO:0098703∼calcium ion import across plasma membrane	8	5.53 × 10^−09^	30.06	4.20 × 10^−06^

As we have seen, many of the genes involved in ion transport have multiple paralogues with tandem exon duplication substitution events. To assess to what extent the presence of multiple genes from the same family (paralogs) may be responsible for the enrichment of GO terms we removed gene duplications choosing a single gene per family and repeated the analysis. The results can be seen in supplementary table S3, Supplementary Material online. Even without the gene duplications, seven of the top ten enriched GO terms, including glutamatergic synapse, membrane, plasma membrane, Z-disc, and extracellular exosome, are still significantly enriched. Terms related to transmembrane ion transport are no longer significantly enriched, however.

### Tandem Exon Duplication Substitution Events in *D. melanogaster*

The overwhelming majority of the 236 human tandem exon duplication substitution events appeared early in the evolution of vertebrates, so most tandem exon duplication substitution events in *D. melanogaster* should be unrelated. We find 154 *D. melanogaster* genes with tandem exon duplication substitution events (supplementary table S4, Supplementary Material online), just over 1.1% of the 13,969 *D. melanogaster* coding genes (FB2020_01). The proportion is similar to that of the human gene set, 214 genes with tandem exon duplication substitution events among 19,988 annotated coding genes (1.07%) in GENCODE v40.

We determined the distribution of GO functional terms for the 154 *D. melanogaster* genes. Results can be seen in table 2. The results are similar to those of the human set. *Drosophila melanogaster* genes with tandem exon duplication substitution events are significantly enriched in “plasma membrane” (9.6 × 10^−11^) and related terms, whereas other terms were also related to those in the human list (muscle contraction, 2.6 × 10^−08^, postsynaptic membrane, 4.5 × 10^−04^). In fact, the first six terms in the *D. melanogaster* list are also significantly enriched in the human gene set.

**Table 2:** Top GO Terms *D. melanogaster* Genes with Tandem Exon Duplication Substitution Events

GO terms	Count	*P*-value	Enrichment	Benjamini
GO:0005886∼plasma membrane	47	4.97 × 10^−13^	3.19	9.59 × 10^−11^
GO:0006936∼muscle contraction	8	3.49 × 10^−11^	52.01	2.58 × 10^−08^
GO:0005509∼calcium ion binding	18	3.77 × 10^−09^	6.19	1.01 × 10^−06^
GO:0005887∼integral component of plasma membrane	25	1.55 × 10^−08^	3.92	1.50 × 10^−06^
GO:0016021∼integral component of membrane	68	1.49 × 10^−07^	1.78	9.57 × 10^−06^
GO:0045211∼postsynaptic membrane	8	9.31 × 10^−06^	10.54	4.49 × 10^−04^
GO:0043005∼neuron projection	10	1.55 × 10^−05^	6.77	5.99 × 10^−04^
GO:0030425∼dendrite	10	6.16 × 10^−05^	5.69	0.002

### Extensive Evidence for Convergent Evolution of Tandem Duplicated Exon Events

Curiously, 54 human genes with tandem exon duplication substitution events (25.2%) have *D. melanogaster* orthologues that also have tandem exon duplication substitution events. These 54 genes (listed in supplementary table S5, Supplementary Material online) can generate 91 distinct tandem exon duplication substitution events between them, but 49 of these events arose from gene duplications. The remaining 42 events belong to 33 gene families. The *D. melanogaster* genes have fewer paralogues with tandem exon duplication substitution events than the human genes—in total 38 *D. melanogaster* tandem exon duplication substitution genes have tandem exon duplication substitution events in human orthologues.

We inspected these 42 events carefully and found evidence of a common ancestry for 7 of the events (Gómez et al. 2021), while the remaining appeared to had evolved in parallel by convergence. Some cases, such as the tropomyosin family (4 genes and 13 events in human, 2 genes and 7 events in fruit fly) have an event with common ancestry and several which evolved independently (Irimia et al. 2010). The 27 orthologous gene families that have unrelated human and *D. melanogaster* tandem exon duplication events are clear examples of convergent evolution in alternative splicing.

The coincidence of events in orthologous genes is considerably more than was detected in a similar analysis of mouse and *D. melanogaster* microexons (Torres-Méndez et al. 2022). In this study, the authors found microexons in 156 *D. melanogaster* genes and 2,731 mouse genes. However, microexons were found in mouse orthologues for just 19 of the genes in the *D. melanogaster* set and none were conserved, even though many microexons are conserved in mammals (Irimia et al. 2014).

The 54 genes with tandem exon duplication substitution events in both species are significantly enriched in the same tissue-specific GO terms (supplementary table S6, Supplementary Material online). Most human genes have undergone gene duplication since the Bilaterian clade, and many maintained the tandem exon duplication substitution events in both copies of the gene. However, even if we only include a single representative for each of the 33 human gene families with tandem exon duplication substitution events in both species, the tissue-specific terms Z-disc (7.6 × 10^−6^) and muscle contraction (0.003) stand out, along with transmembrane calcium ion transport terms (calcium ion import across plasma membrane, 0.003; calcium ion transmembrane transport, 0.006).

### Enrichment in Tandem Duplicated Exon Events in Voltage-Gated Ion Channels

Many of the human and *D. melanogaster* genes with tandem exon duplication substitution events are involved in transmembrane ion transport. A total of 27 of the 154 *D. melanogaster* genes (17.5%) and 42 of 214 human genes (19.6%) have this function. Although there are no GO terms for “ion transport” or “ion channel”, there are UniProtKB keywords (The UniProt Consortium 2021), and both terms are highly enriched among human and *D. melanogaster* genes with tandem exon duplication substitution events. Ion transport would be the most enriched term in human genes (4.2 × 10^−12^) and would be sixth in *D. melanogaster* (1.7 × 10^−05^) list. The UniProtKB keyword “ion channel” had Benjamini values of 1.39 × 10^−^0^9^ in human and 4.6 × 10^−06^ in *D. melanogaster*.

Among these genes, the voltage-gated ion channels are particularly enriched. In the human gene set, there are 105 genes in 14 voltage-gated ion channel families, 61 of which from 8 voltage-gated potassium channel families. *Drosophila melanogaster* has just 16 genes in 13 voltage-gated ion channel families. Several families of voltage-gated ion channel genes (Moran et al. 2015) have evolved tandem duplicated exon regions independently in human and *D. melanogaster*. There are tandem exon duplication substitution events in 4 of the 14 human voltage-gated ion channel families and in 5 of the 13 *D. melanogaster* families.

Although genes with tandem exon duplications are clearly enriched across all voltage-gated ion channels, one superfamily, which combines the three voltage-gated calcium channel families and the voltage-gated sodium channel family, stands out. The human gene set has 20 genes, 10 voltage-gated sodium channel genes and 10 calcium channel genes. Eleven human voltage-gated channel genes have tandem exon duplication substitution events, though most derive from gene duplications, *D. melanogaster* has just four genes in this superfamily, one for each family, and all four have tandem exon duplication substitution events.

**Fig. 4:**
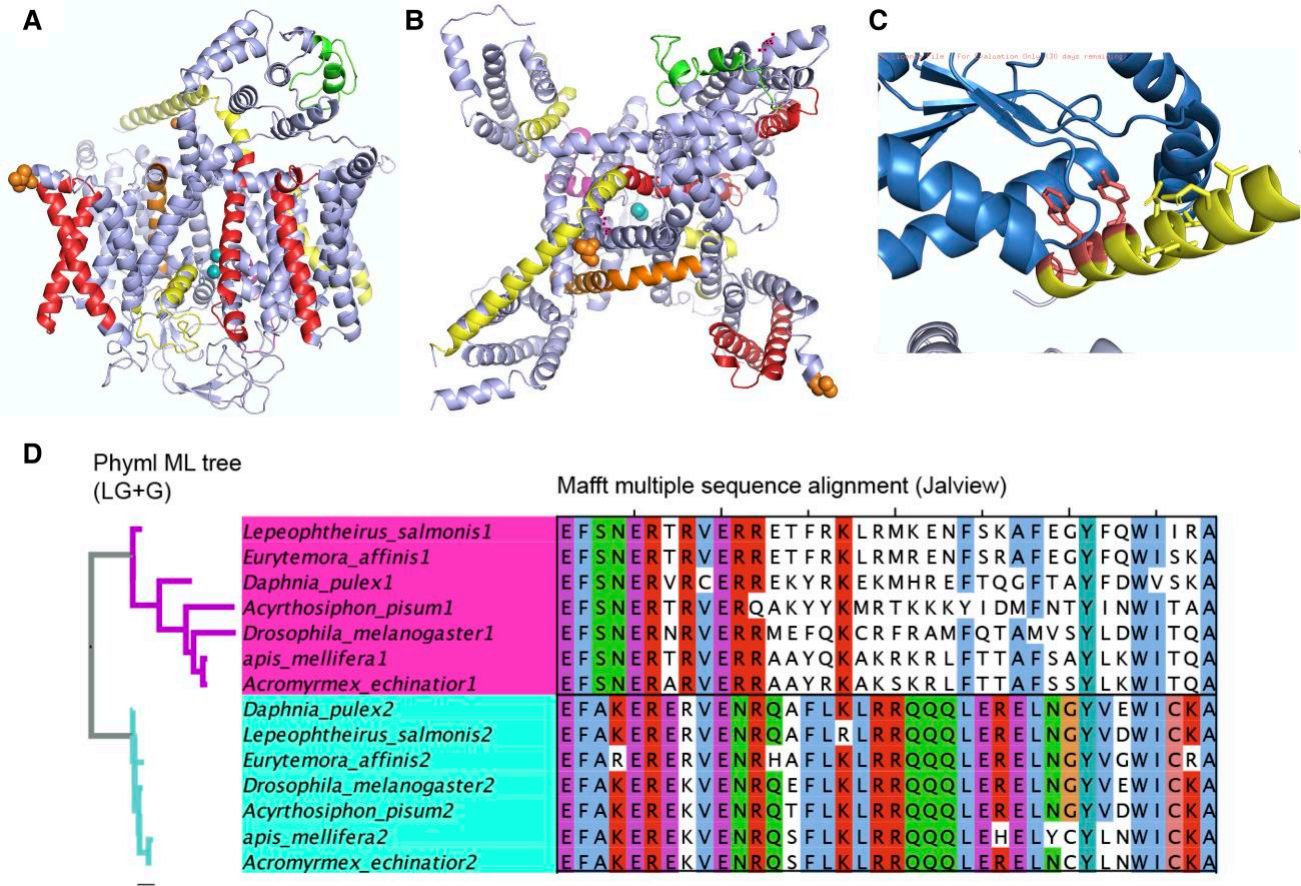
CACNA family homologous exons in *Drosophila melanogaster* and human. Tandem exon duplication substitution events mapped onto the structure of rabbit *CACNA1S* (PDB: 5gjv) and colored by gene family. Panel *A*: side view of *CACNA1S* showing the helices that cross the membrane. Yellow regions are from *D. melanogaster* gene *Cacophony*, orange regions from *D. melanogaster* gene *Ca-alpha1D*, magenta from *D. melanogaster* gene *Ca-alpha1T*, red from human genes *CACNA1C* and *CACNA1D*, and green from human genes *CACNA1A*, *CACNA1B*, and *CACNA1E*. Panel *B*: top view of the transporter showing the transmembrane pore. Colors identical to *A*. Panel *C*: a single tandem exon duplication substitution region mapped onto the structure of rabbit *CACNA1S*. The structure colored in blue is the protein from *CACNB1*, which interacts with one of the regions coded by a tandem exon duplication substitution event in *D. melanogaster* gene *Cacophony*. Panel *D*: cross-species alignments of regions coded by the tandem exon duplication from arthropod species for the tandem exon duplication substitution event in section *C*.

The voltage-gated calcium channels are involved in a variety of processes, including muscle contraction and neurotransmitter release. In total, the 13 human and *D. melanogaster* voltage-gated calcium channel genes have 20 tandem exon duplication substitution events between them (fig. 4). In human, L-type calcium channels (*CACNA1C* and *CACNA1D*) have three events each, and the P/Q, N and R-type calcium channels (*CACNA1A*, *CACNA1B*, and *CACNA1E*) have a single event each. The *D. melanogaster* reference set has three genes, *Ca-alpha1D* (with two tandem exon duplication substitution events), *Ca-alpha1T* (a single event), and *Cacophony* (three distinct tandem exon duplication substitution events). In total, there have been ten independent tandem exon duplication substitution events in human and *D. melanogaster* voltage-gated calcium channels, and all ten events affect different exons (fig. 4).

One tandem exon duplication in *Cacophony* is particularly interesting—it codes for a region that in the rabbit homologue *CACNA1S* is crucial for the interaction with *CACNB1* (fig. 4*C*). The event in *Cacophony* covers 39 amino acid residues, and 21 of these are conserved between the more similar homologous exon in *Cacophony* and human orthologue, *CACNA1S*. The conservation of the region between the human and *D. melanogaster* genes strongly suggests that this region binds *CACNB1* (or its *D. melanogaster* equivalent, *Ca-beta*) across vertebrate and invertebrate voltage-gated calcium channels. The event in *Cacophony* is conserved across invertebrate species. We found evidence of both tandem duplicated exons in insects (flies and ants) and in crustaceans (crabs, *Daphnia*), showing that the exon duplication is at least 530 million years old (fig. 4*D*). This reinforces the importance of this tandem exon duplication.

Although the region is highly conserved between vertebrates and invertebrates, just 16 of the 39 amino acid residues are conserved between the two distinct *Cacophony* isoforms and many of the amino acid replacements are not conservative. Given that this region is important for the interaction with *Ca-beta* (the *D. melanogaster CACNB1* orthologue), one hypothesis would be to imagine that the alternative isoform of *Cacophony* binds a different protein. However, closer inspection shows that the unchanged residues in duplicated exons are those that would interact most closely with *Ca-beta*, while the most drastic residue changes are on the inside of the helix (away from the interaction with *Ca-beta* and not in close contact with the rest of the protein). In particular, the tryptophan that buries itself deep into the binding cleft in Ca-beta, its flanking isoleucine and a tyrosine that is also important for binding are maintained between the two isoforms and in all invertebrate species. The evidence suggests that both isoforms bind *Ca-beta* and that the tandem exon duplication may moderate the effect of *Ca-beta* binding on *Cacophony*.

There is a tandem exon duplication substitution event in a voltage-gated calcium channel family that is even older than the event in *Cacophony*. The single substitution event in *Ca-alpha1T* is conserved across arthropods and is present in the *Caenorhabditis elegans* orthologue (*cca-1*) and other nematode species. In fact, the two exons are even found in molluscs (supplementary fig. S2, Supplementary Material online), so the event is conserved across protostomes. It evolved more than 610 million years ago, but was apparently lost prior to the chordate lineage.

There is documented evidence for the functional consequences of tandem duplicated exon events in several voltage-gated ion channels. One of the three tandem exon duplication events in *D. melanogaster* gene *Slowpoke* (*Slo*) produces isoforms that differ in calcium sensitivity, while another event generates differences in conductance (Lagrutta et al. 1994). In the German cockroach, the tandem duplicated exon event in the voltage-gated sodium channel gene *para* produces isoforms with different sodium gating properties (Tan et al. 2002).

Physiological differences have also been recorded for other voltage-gated channels, for example, *CACNA1B* and *NALCN*. Tandem duplicated exon events in mouse *Cacna1b* produce brain and nociceptor-specific isoforms. It has been shown that the nociceptor isoform affects complex behavior by enhancing responses to adverse stimuli (Bunda et al. 2019). The sodium leak channel gene *NALCN* has two known tandem duplicated exon events that are found in a range of invertebrate species (though not in *D. melanogaster*). The tandem duplicated exon events generate isoforms with distinct residue patterns that are selective for either calcium or sodium ions (Senatore and Spafford 2013).

### Tandem Duplicated Exon Substitution Events in Nematodes

**Fig. 5:**
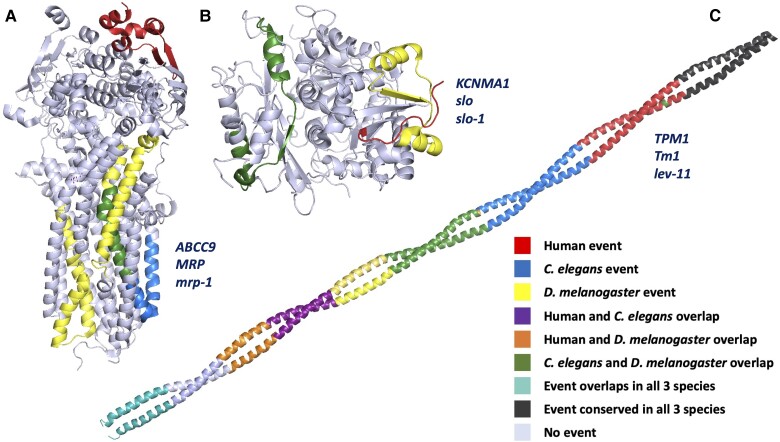
Tandem duplicated exon events in *Caenorhabditis elegans*, *Drosophila melanogaster*, and human. Events are coloured by species as per the legend. (*A*) Tandem exon duplication substitution events for *ABCC9*, and *D. melanogaster* and *C. elegans* orthologues (*mrp* and *mrp-1*), mapped onto the structure of human cystic fibrosis transmembrane conductance regulator (6o1v) colored by species. *mrp* and *mrp-1* have unrelated events that coincide in the same region of the structure. The event unique to *mrp* has eight interchangeable homologous exons. (*B*) Tandem exon duplication substitution events mapped onto the structure of human *KCNMA1* (3naf). Both *D. melanogaster slo* and *C. elegans slo-1 have unrelated events that coincide in the same region of the structure*. (*C*) Tandem exon duplication substitution events mapped onto the structure of human *TPM1* (1c1g). *D. melanogaster Tm1* and *C. elegans lev-11* have apparently non-orthologous tandem exon duplication substitution events, as do *TPM1* and *Tm1*, and *TPM1* and *lev-11*. *TPM1*, *lev-11*, and *Tm1* all have three tandem duplicated 3' exons that can produce homologous C-terminals, but the exons appear not to have evolved from a common ancestor. A further unrelated tandem exon duplication substitution generates distinct C-terminals in *D. melanogaster Tm2*. Only the N-terminal tandem exon duplication substitution event seems to be conserved between the three species. In this event, the first two exons are swapped for a homologous 5′ exon which produces a shorter isoform. This event evolved in a common ancestor almost 700 million years ago. Only one exon (the penultimate) is not involved in a tandem exon duplication substitution event in at least one of the three species. Mapping to 3D structures was carried out using HHPred (Zimmermann et al. 2018).

Not only is there a surprising number of gene families with tandem exon duplication substitution events in both vertebrate and arthropod species, but a number of these gene families also have nematode orthologues with unrelated tandem exon duplication substitution events. For example, *ABCC9* (Yue et al. 2017) and *KCNMA1* have distinct homologous exon events in human, *D. melanogaster* and *C. elegans* orthologues (fig. 5). In tropomyosin genes (Irimia et al. 2010), one tandem exon duplication substitution (at the N-terminal) is conserved across arthropods, nematodes, and vertebrates, but the three species have further unrelated tandem exon duplication events. There are another four tandem exon duplication substitution events in human tropomyosin genes, *D. melanogaster* tropomyosin genes have another three, and *C. elegans* gene *lev-11* has six more events. None, apart from the N-terminal event, appear to be related (fig. 5).

In addition to these three genes, at least nine other orthologous genes or gene families are annotated with tandem exon duplication substitution events in vertebrates, arthropods, and nematodes. These are the actinin gene family (*Actn* in *D. melanogaster* and *atn-1* in *C. elegans*), *AKR1C3* (*CG10638* and *Y39G8B.1*), *ATE1* (*Ate1* and *Ate-1*), the Plasma membrane calcium-transporting ATPase gene family (*PMCA*, *mca-3*), *GALNT13* (*Pgant5*, *gly-5*), *GLRA2* (*GluClAlpha*, *avr-14*), *PRKCB* (*Pkc53E*, *pkc-2*), *RYR3* (*Ryr*, *unc-68*), and the sodium channel subunit alpha gene family (*para*, *cca-1*).

This clear evidence of convergent evolution strongly suggests that tandem exon duplication substitution events are intrinsically linked to certain functions. Tandem exon duplication substitution events in certain genes may also have played important evolutionary roles. Six of the 12 gene families with tandem exon duplication substitution events across arthropod, nematode, and vertebrate species are involved in transmembrane ion transport.

## Discussion

The mutually exclusive alternative splicing of tandem duplicated exons gives rise to polypeptide regions that are distinct, but homologous. This allows genes to produce two (or more) proteins that are identical save for a (generally) short region of homologous sequence. These tandem duplicated exon substitution events are rare, there are just 236 annotated in the current GENCODE human gene set, and we find just 154 in *D. melanogaster*. Almost a quarter of the human genes have a *D. melanogaster* orthologue that also has tandem duplicated exon substitution events, most of which appear to have evolved independently. This suggests that certain gene families can particularly benefit from the functional innovation brought about through the mutually exclusive splicing of tandem duplicated exons.

Despite their infrequency, tandem duplicated exon substitution events appear to have much more functional relevance than all other splice event types. The vast majority are of ancient origin (Kondrashov and Koonin 2001; Abascal, Tress, et al. 2015; Gómez et al. 2021), many are detectable in proteomics experiments (Abascal et al. 2015; Rodriguez et al. 2020; Gómez et al. 2021), and they have proportionally 25 times as many pathogenic mutations as all other alternative exons (Gómez et al. 2021). Mutually exclusively spliced tandem duplicated exons make up almost a third of alternative exons that can trace their ancestry back to fish (Gómez et al. 2021). Close to two-thirds of the 236 events annotated in the human genome were already present in the ancestor of vertebrates, more than 500 million years ago and more than 90% have orthologous events in teleost fish (Gómez et al. 2021).

Here, we analyzed in detail the UHP regions produced by the set of 236 tandem exon duplication-derived substitution events annotated in GENCODE v40. We find that C-terminal and N-terminal UHP regions are highly enriched. The enrichment in terminal UHP regions means that UHP regions often coincide with transmembrane helices, coiled coils, and disordered regions. Within all UHP regions, most residue changes between UHP regions are conservative, rather than drastic, and core structural residues and those that bind small ligands are significantly more conserved. This suggests that selection is acting on the duplicated exons to limit the changes to the structure and function of the UHP regions.

An extraordinarily high proportion of UHP regions are tissue-specific in proteomics experiments. The proportion of UHP regions that are tissue-specific in at least one tissue or tissue group is double what was found in a previous analysis (Rodriguez et al. 2020). For those events with a common ancestor in humans and teleost fish, more than 75% of UHP regions were tissue-specific. By way of contrast, primate-derived exons, which make up 75% of alternative exons, tend not to produce tissue-specific isoforms (Rodriguez et al. 2020).

Both human and *D. melanogaster* genes with tandem duplicated exon substitution events are highly enriched in nervous and muscle-specific functional terms, and almost half produce proteins with at least one transmembrane helix. Human and *D. melanogaster* genes with tandem duplicated exon substitution events are significantly enriched in ion channels and ion transport. In fact, genes from 9 of the 33 human and *D. melanogaster* families that have tandem duplicated exon substitution events are involved in transmembrane ion transport, as are 6 of the 12 families with tandem duplicated exon substitution events in arthropods, nematodes, and vertebrates. The evidence for the incorporation of tandem duplicated exon substitution events appears in genes involved in transmembrane ion transport strongly suggests that there is some sort of adaptive benefit from tandem duplicated exon substitution events in ion transport, and in particular in ion channels (Copley 2004).

Research implicates transmembrane ion transporters in tissue and organ development (Levin 2014; Liebeskind et al. 2015; Hegedus et al. 2020; Chen et al. 2021). Given the ancient origin of the vast majority of tandem duplicated exon substitution events (Gómez et al. 2021), the coincidence of tandem duplicated exon substitution events in orthologous vertebrate and invertebrate gene families, the extraordinarily high levels of tissue specificity in proteomics experiments, and the enrichment in tissue-specific functional terms, it is not too much of a stretch to hypothesize that tandem duplicated exon substitution events may have played important roles in the evolution of metazoan organs and tissues.

## Materials and Methods

### Annotation Databases

We used the GENCODE v40 human gene set (Frankish et al. 2021) as the base for all analyses. Homology searches were carried out against other vertebrate species using the Ensembl (Howe et al. 2021), RefSeq (O'Leary et al. 2016), and UniProtKB (The UniProt Consortium 2021) annotations for those species. We looked for *D. melanogaster* tandem duplicated exon substitution events, and for homology to the human tandem duplication events in FlyBase (Thurmond et al. 2019), and used UniProtKB and APPRIS (Rodriguez et al. 2022) for other invertebrate species.

Alternative exons in the GENCODE v40 human gene set were defined as all exons that did not overlap exons that produced the main isoforms. Main protein isoforms for the GENCODE v40 human gene set were the APPRIS principal isoforms for this release. APPRIS defines principal isoforms based on cross-species conservation and conserved protein features (Rodriguez et al. 2013).

### Tandem Duplicated Exon Substitution Events from the Human Gene Set

We had previously manually curated 236 tandem exon duplication substitution events in 215 genes from the Ensembl/GENCODE human gene set (Frankish et al. 2021). The manual curation was carried out over a 6-year process and is described in detail in a previous paper (Gómez et al. 2021). We made small changes to the set for this paper. We removed one tandem exon duplication (from gene *TNC*) because we no longer believe that it is mutually exclusively spliced, and we added an event that was not previously annotated in the Ensembl/GENCODE reference set, but has been included to the GENCODE v40 gene set, a third mutually exclusively spliced exon in *SLC12A1* (Payne and Forbusch 1994). The full set of human tandem exon duplication substitution events we used in this analysis had 236 events across 214 coding genes.

### Tandem Duplicated Exon Substitution Events from the *D. melanogaster* Gene Set

Given that almost all tandem exon duplication substitution events were present in the ancestors of teleost fish, dating their origins to more than 425 million years ago. Most differences with vertebrate species are likely to be due to gaps in the annotation of non-human species. However, a comparison with *D. melanogaster* might shed light on the functional importance of tandem exon duplication substitution events. *D. melanogaster* is well annotated (Thurmond et al. 2019) and just seven tandem exon duplication substitution events have evidence of conservation between the *D. melanogaster* and human gene sets.

We manually annotated as many tandem exon duplication substitution events as we could find from the *D. melanogaster* gene set (Flybase version: FB2022_2). The tandem exon duplication substitution events came from a range of sources (Kondrashov and Koonin 2001; Tress et al. 2008; Hatje and Kollmar 2013; Gómez et al. 2021; Rodriguez et al. 2022). As with the tandem exon duplication substitution events, we did not take the predictions of homology at face value. The translated UHP regions each had to have at least eight amino acid residues. The UHP regions had to either be detected in a BLAST search (Altschul et al. 1990) with an e-value of 0.01, or to have other evidence of homology. For example, both UHP regions might be most similar to the same 3D structure, or map to the same functional domain or motif.

We found 154 tandem exon duplication substitution events in the *D. melanogaster* gene set.

### Proteomics

We analyzed two large-scale proteomics analyses (Kim et al. 2014; Wang et al. 2019) for evidence of the translation of tandem exon duplication substitution events. These experiments were carried out on multiple tissue types. We downloaded the data from ProteomeXchange (Deutsch et al. 2017) with the identifiers PXD000561 and PXD010154. The two experiments interrogated 43 distinct tissues and cell types.

We used the GENCODE human reference set for the peptide search database, excluding read-through genes (Abascal et al. 2018) because these genes are not coding and would disrupt the peptide mapping algorithm. We set aside those experiments that did not use trypsin to limit the false positive matches. Even though other enzymes can theoretically detect different peptides (Wang et al. 2018), they are less specific than trypsin, and tryptic peptides are ideally suited for detection in standard proteomics experiments (Hildonen et al. 2014). In any case, allowing missed cleavages by trypsin substantially improves alternative splicing coverage without the disadvantages of non-tryptic enzymes.

We mapped spectra from the two experiments to the GENCODE gene set using the Comet search engine (Eng et al. 2013). Comet was run with default parameters, allowing oxidation of methionines. Peptide spectrum matches (PSM) were post-processed using Percolator (The et al. 2016). Percolator posterior error probability (PEP) values were used to filter Comet PSM. Peptides that passed the filters were required to be fully tryptic and to have a maximum of one missed cleavage. Peptides that mapped to more than one gene were discarded.

Combining spectra from many different experiments will inevitably expand the false discovery rate (Ezkurdia et al. 2015b). We used a conservative PEP score of 0.001 to filter the peptides. To reduce false positive identifications further, each peptide had to be identified in at least two different experiments. These steps will have eliminated many false positive matches; we found no peptides that mapped to olfactory receptors (e.g., Ezkurdia et al. 2014). In the case of tandem exon duplication substitution events, the protein sequences of the two UHP regions will have different amino acid sequences. To detect the splice event, we required that both UHP regions were validated by distinct peptides (Rodriguez et al. 2020).

### Tissue Specificity Calculations

We are analyzing large-scale, label-free proteomics experiments (Kim et al. 2014; Wang et al. 2019) for a large range of tissues, the majority of which have multiple, replicate experiments. As a proxy for quantification, we counted the number of distinct experiments in which a peptide was detected. Tissue specificity was confirmed as follows. First, for each peptide that distinguished a UHP region, we counted up the number of distinct experiments the peptide was detected in, both in total and within each tissue. These PECs were used to determine whether a UHP region was tissue-specific. For each tissue and each tandem exon duplication substitution event, we carried out a Fisher's exact test comparing the PECs for peptides that uniquely discriminated one UHP region, with the PECs for peptides that distinguished the other UHP region. For the tissue-specificity analysis, both UHP regions had to be supported by a minimum of three PECs, because it is impossible to detect tissue specificity in events supported by fewer than three PECs. Tissue-specific events were those that had significantly elevated PECs for one UHP region.

### Evidence for Tissue-Specific Splicing in Proteomics Experiments

We also collected tissues into 13 different groups. The relationship between groups and tissues are shown in supplementary table S1, Supplementary Material online. Grouping the tissues in this way allowed us to expand the detection of tissue-specific events, since events could either be tissue-specific or tissue-group specific.

### Physicochemical Characteristics

We downloaded the EBI-AlphaFold models (Varadi et al. 2022) for the UniProtKB display sequences. In total, there were 23,432 models, in large part because AlphaFold has to model proteins in chunks of 2,000 residues. Titin is modeled in 166 chunks, for example. The models corresponded to 20,504 UniProtKB entries. UniProtKB entries are different from GENCODE genes for a number of technical and historical reasons (Abascal et al. 2018), and there are 1,143 UniProtKB proteins that were not annotated in GENCODE. It is likely that some of the differences between UniProtKB and GENCODE is due to SNPs.

We calculated disorder as per the suggestion of the AlphaFold authors. Residues that had a pLDDT reliability index of 50 or less were predicted to be disordered. We calculated the disorder for the UHP regions in genes with tandem duplicated exon events based on the UHP regions in the models. The models based on the display variants had neither of the UHP regions in five of the 236 events (*KCNAB2*, *KCNIP4*, *RGPD1*, *SPAG11A*, and *SPAG11B*), and included both UHP regions in four events (*ITGA6*, *ITGA7* and two in *CACNA1C*). We left the first five genes out of the analysis of disordered residues. In the case of the events in *ITGA6*, *ITGA7*, and *CACNA1C*, one of the UHP regions was predicted to have few or no disordered residues, whereas the other was mostly disordered. In these cases, the second UHP region is only disordered because it does not belong in the principal isoform, so we removed the disordered UHP region and maintained the UHP region with few disordered residues.

Coiled coil and transmembrane regions were detected by viewing the models and cross-checking with UniProtKB, APPRIS, and Pfam (El-Gebali et al. 2019) annotations, and checking against the known literature.

### Accessibility to Solvent

Secondary structure and relative solvent accessibility (RSA) estimation of the set of tandem exon duplication substitution events were calculated using the Dictionary of Protein Secondary Structure (DSSP; Kabsch and Sander 1983) module available in Biopython (Cock et al. 2009). The DSSP program calculates the most likely secondary structure assignment by reading the position of the atoms in a protein followed by the calculation of the H-bond energy between all atoms. The RSA of an amino acid residue is defined as the ratio of the solvent-exposed surface area of that residue observed in a given structure and the maximum obtainable value of the solvent-exposed surface area for this amino acid. Thus, RSA adopts values between 0 and 1, with 0 corresponding to a fully buried and 1 to a fully accessible residue, respectively.

We removed the predicted disordered residues for the analysis (35,444 out of 166,794) and collapsed the eight types of secondary prediction into three main types (coil/loop, strand, and helix).

### Functionally Important Residues

Ligand binding and catalytic site residues were predicted for the 214 genes in the set using the *firestar* web server (Lopez et al. 2011). *Firestar* predicts ligand binding sites based on homology to known structures that bind ligands. *Firestar* predicted ligand binding residues for 95 genes that have tandem exon duplication substitution events. Ligand binding residues coincided with the UHP regions in 26 of these events.

### Gene Ontology (GO) Terms

Gene Ontology (The Gene Ontology Consortium 2019) terms for the 214 genes with tandem duplicated exon substitutions in the human gene set and for the 154 genes with tandem duplicated exon substitutions in the *D. melanogaster* gene set were predicted using the web tool, DAVID (Sherman et al. 2022).

## Supplementary Material

evac162_Supplementary_Data

## Data Availability

There are no new data associated with this article. The data sets were derived from sources in the public domain: the AlphaFold models from the EBi-AlphaFold collaboration (https://alphafold.ebi.ac.uk/), the *D. melanogaster* annotations are originally from FlyBase (https://flybase.org/), the human gene set annotations are from GENCODE (https://www.gencodegenes.org/human/), and the proteomics data sets from ProteomeXchange (https://www.proteomexchange.org/).
