# Supplementary material for: Origins and Evolution of Human Tandem Duplicated Exon Substitution Events
Source: Genome Biol Evol. 2022 Nov 8;14(12):evac162. doi: 10.1093/gbe/evac162 (PMC9741552; doi:10.1093/gbe/evac162)
Supplement: evac162_Supplementary_Data [file evac162_supplementary_data.zip › SupplFigs.docx]

**
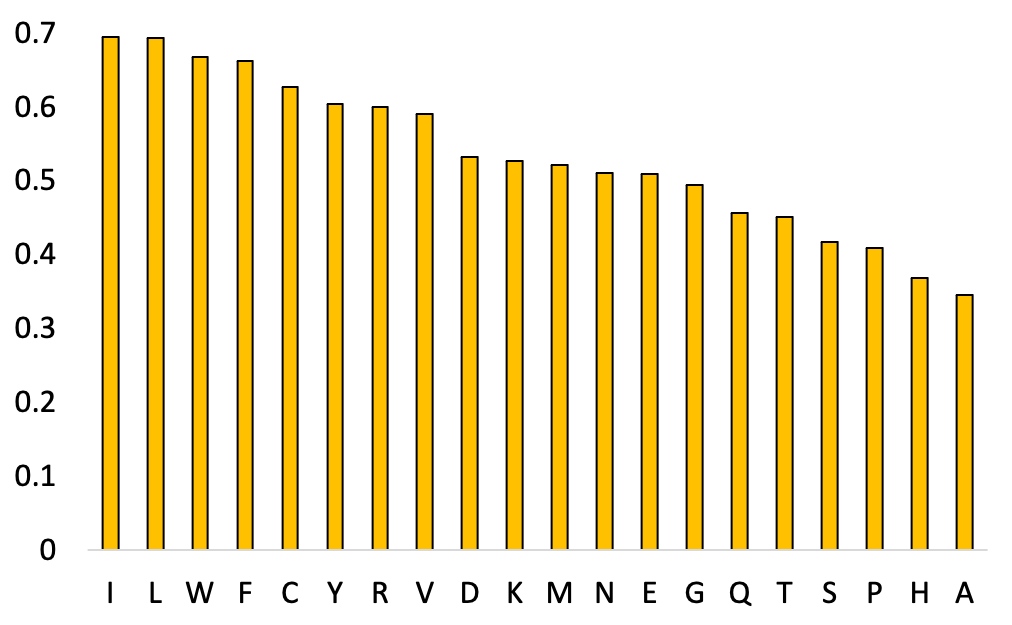
**

**SFigure 1. The relative conservation of amino acids in UHP regions**

If we take a McLachlan matrix score of 5 as being a highly conservative change, more than two thirds of isoleucine, leucine and tryptophan residues are either conserved, or have a highly conservative change in the alignments between UHP regions, and the next three amino acids that undergo least physicochemical change are phenylalanine, cysteine and tryptophan. As a comparison, just over a third of alanine residues (34.4%) are conserved.


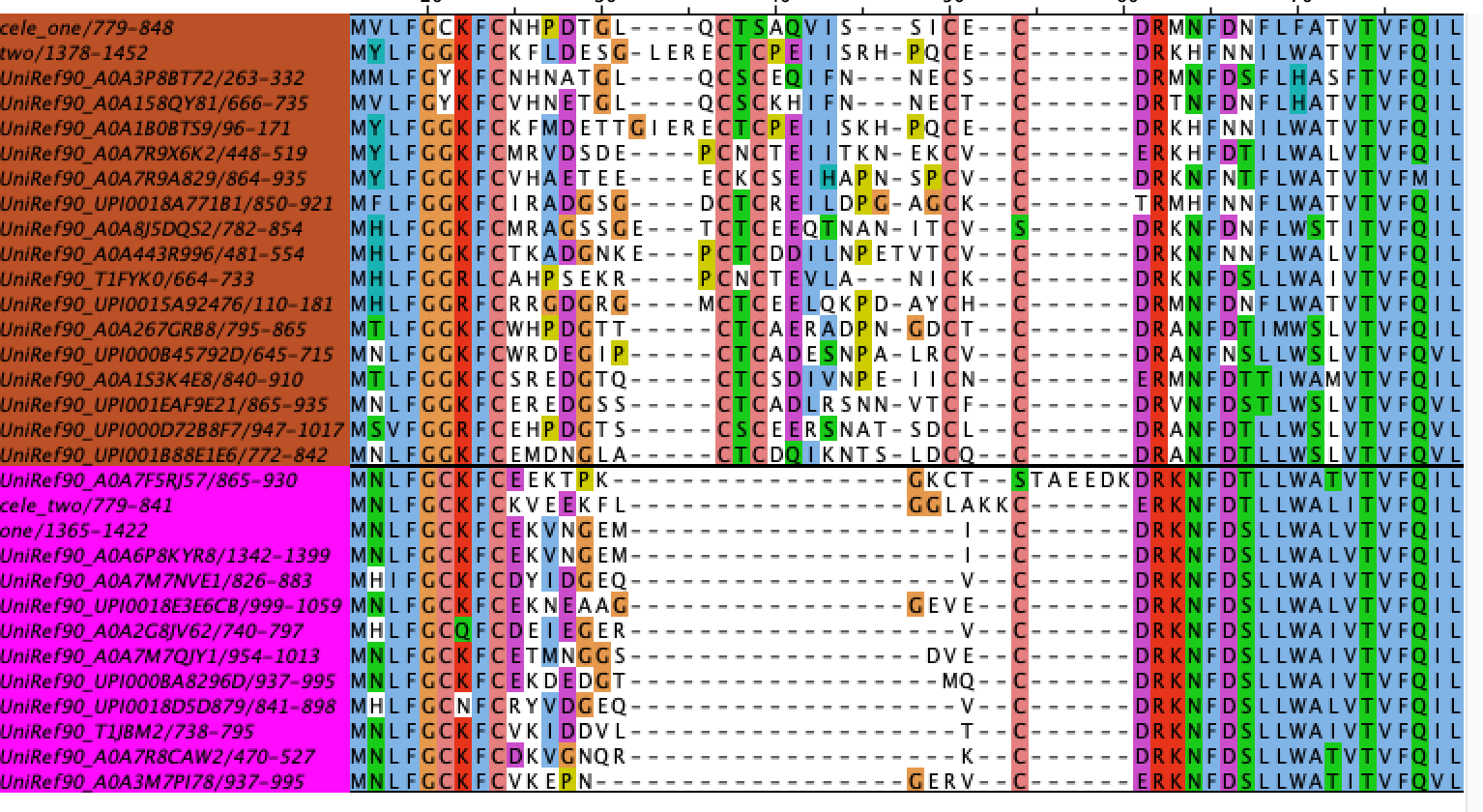


**SFigure 2. The alignment between *C-alpha1T* UHP regions in invertebrate species**

Alignments over the region translated from C-alpha1T tandem duplicated exon substitutions in UniRef90 sequences from invertebrate species. Sequences corresponding to the one of the two UHP regions are brown, sequences that correspond to the other UHP region in pink.
